# Glucose and Auxin Signaling Interaction in Controlling *Arabidopsis thaliana* Seedlings Root Growth and Development

**DOI:** 10.1371/journal.pone.0004502

**Published:** 2009-02-18

**Authors:** Bhuwaneshwar S. Mishra, Manjul Singh, Priyanka Aggrawal, Ashverya Laxmi

**Affiliations:** National Institute for Plant Genome Research, Aruna Asaf Ali Marg, New Delhi, India; Cairo University, Egypt

## Abstract

**Background:**

Plant root growth and development is highly plastic and can adapt to many environmental conditions. Sugar signaling has been shown to affect root growth and development by interacting with phytohormones such as gibberellins, cytokinin and abscisic acid. Auxin signaling and transport has been earlier shown to be controlling plant root length, number of lateral roots, root hair and root growth direction.

**Principal Findings:**

Increasing concentration of glucose not only controls root length, root hair and number of lateral roots but can also modulate root growth direction. Since root growth and development is also controlled by auxin, whole genome transcript profiling was done to find out the extent of interaction between glucose and auxin response pathways. Glucose alone could transcriptionally regulate 376 (62%) genes out of 604 genes affected by IAA. Presence of glucose could also modulate the extent of regulation 2 fold or more of almost 63% genes induced or repressed by IAA. Interestingly, glucose could affect induction or repression of IAA affected genes (35%) even if glucose alone had no significant effect on the transcription of these genes itself. Glucose could affect auxin biosynthetic YUCCA genes family members, auxin transporter PIN proteins, receptor TIR1 and members of a number of gene families including AUX/IAA, GH3 and SAUR involved in auxin signaling. *Arabidopsis* auxin receptor *tir1* and response mutants, *axr2*, *axr3* and *slr1* not only display a defect in glucose induced change in root length, root hair elongation and lateral root production but also accentuate glucose induced increase in root growth randomization from vertical suggesting glucose effects on plant root growth and development are mediated by auxin signaling components.

**Conclusion:**

Our findings implicate an important role of the glucose interacting with auxin signaling and transport machinery to control seedling root growth and development in changing nutrient conditions.

## Introduction

All organisms need to be able to sense and respond to the changing nutrients status, such as availability of sugars. Plants, being sessile, especially need to be able to adapt to changing availability of nutrients in the environment. As a result, a number of plant developmental, physiological and metabolic processes are regulated in response to changing levels or flux of soluble sugars. Sugars have affect on almost all phases of plant life cycle from seed germination to hypocotyl elongation, cotyledon expansion, adventitious root formation, true leaf formation, flowering and senescence [1], [2]. Recent studies have also provided significant evidence of interactions between sugar and phytohormone response and other metabolic pathways [3]–[5]. Among the phytohormones, auxin is very important for plant growth and development. Auxin can also stimulate cell division and cell elongation. It also controls lateral and adventitious root formation and mediates the tropic response to gravity and light. Auxin promotes flowering, delays leaf senescence, fruit ripening and can inhibit or promote leaf and fruit abscission. Since a number of common responses are regulated by sugar and auxin, the obvious question arising is whether sugar and auxin act independently or interdependently to bring about changes in plant development and morphology/architecture. Although, both sugar and auxin are so fundamental to plants and regulate similar processes, yet no systematic study has been done to explore the molecular bases of interaction between these two signalling molecules. There are only very few reports in *Arabidopsis* providing evidence that these two signaling pathways interact with each other. Glucose insensitive mutant *gin2*, which is mutated in glucose sensor *HXK* gene, is also resistant towards exogenous auxin application [6]. Another mutant, turanose insensitive (*tin*), encodes for WOX5 gene, which is responsible for auxin homeostasis and maintaining auxin maxima in the root tip [7]. Another, very recent report is about a mutant allele of *hls1* (n-acetyl transferase) which is perturbed in both sugar and auxin responses [8]. Here, whole genome approach has been taken up to accomplish a thorough analysis of nature of interaction between these two signalling pathways in a model plant system *Arabidopsis thaliana* using root growth and development as a tool.

## Results

**Figure 1:**
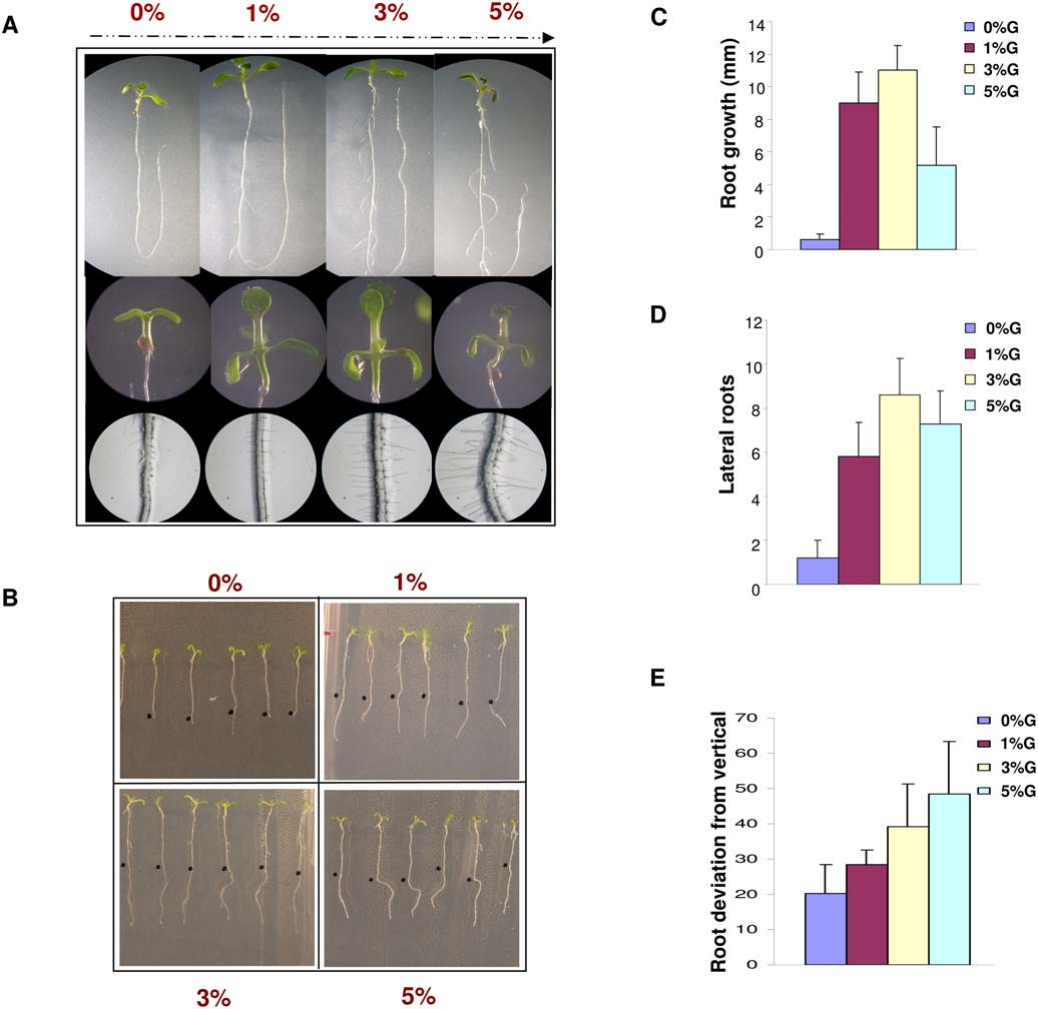
Comparison of root growth and development of Col seedlings grown in different concentrations of glucose containing medium. (A) Root growth, lateral roots, seedling morphology and root hair formation of 5 d old Col seedlings transferred to increasing concentrations of glucose containing medium for 2–5 days. (B) 5 d old light-grown Col seedlings root angle deviation from vertical increases on transferring them to increasing concentration of glucose containing medium for 2 d. (C) Comparative graph of root length of 5 d old Col light-grown seedlings shifted to different concentration of glucose containing medium for 2 d. The root length increases on increasing glucose concentration up to 3% but decreases if the concentration is increased to 5% or more. Average of 10 seedlings was taken and error bar represents standard deviation. (D) Comparative graph of lateral roots of 5 d old Col light-grown seedlings shifted to different concentration of glucose containing medium for 5 d. Number of lateral roots increase on increasing glucose concentration up to 3% but decrease if the concentration is increased to 5% or more. Average of 10 seedlings was taken and error bar represents standard deviation. (E) Comparative graph of root angle deviation from vertical of 5 d old Col light-grown seedlings shifted to different concentration of glucose containing medium for 2 d. Root angle deviation of Col seedlings from vertical increases on increasing glucose concentrations. Average of 10 seedlings was taken and error bar represents standard deviation.

There are several reports that sugar can influence/modulate plant root length or number of adventitious roots. Interestingly, in our experiments, we observed that increasing concentration of glucose not only increases root length, number of lateral roots and root hair but also modulates gravitropic response of the primary roots of young seedlings. The 5-d-old light-grown Col seedlings shifted to ½ Murashige and Skoog (MS) medium containing various concentrations of glucose displayed not only a change in root length, number of lateral roots, root hair but also the direction of the roots gets more randomized (Figure 1). Presence of 3-O-methylglucose (3-OMG) (non-signaling glucose analog) in the medium could not affect root length, lateral roots and root gravitropism as extensively as is caused by glucose suggesting glucose specificity rather then the osmotic effects to be responsible for these responses (Figure S1).

**Figure 2:**
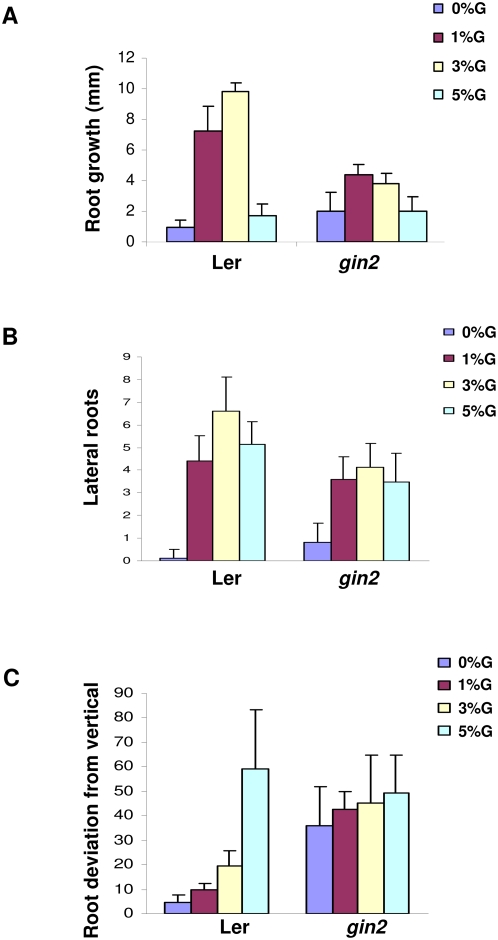
Comparison of root growth and development of Ler and *gin2* seedlings grown in different concentration of glucose containing medium. Comparative graph of root growth (A), lateral roots (B) and root angle deviation (C) of 5 d light grown *gin2* mutant transferred to different concentration of glucose containing medium for 2 d for root length and root deviation measurements and 5 d for lateral root measurements. *gin2* mutant display resistance to changes in root length, lateral root and root deviation on increasing concentrations of glucose as compared to Ler seedlings. Average of 10 seedlings was taken and error bar represents standard deviation.

To check if this is metabolic or signaling effect of glucose, the effect of increasing concentration of glucose on *gin2* (glucose receptor mutant) [6] mutant was checked. The *gin2* (Hexokinase) mutant showed a differential response with respect to change in root length, number of lateral roots and deviation of roots from vertical as compared to Ler grown under similar conditions, suggesting these responses to be dependent on hexokinase mediated signaling. The extent of dependence was different for different responses with lateral root induction less affected then root elongation and deviation from vertical (Figure 2). The *gin2* mutant showed a constitutive phenotype in terms of root deviation from vertical suggesting optimal glucose signaling to be very important for controlling this response.

Since root growth and development is dependent on auxin physiology, the effect of glucose on auxin signaling was further studied by microarray analysis. Light-grown 5 d old Col seedlings grown in 1/2MS medium containing 0.8% agar and 1% sucrose were depleted of sugars by placing them in sugar free 1/2MS liquid medium in dark for 24 h. The seedlings were then treated in dark for 3 h with 1/2MS liquid medium containing different concentrations of glucose and IAA alone and in different combinations (0%G, 0%G+1 µM IAA, 3%G, 3%G+1 µM IAA). RNA was extracted and microarray analysis was done. Microarray data was analyzed using data analysis software ArrayAssist.

**Figure 3:**
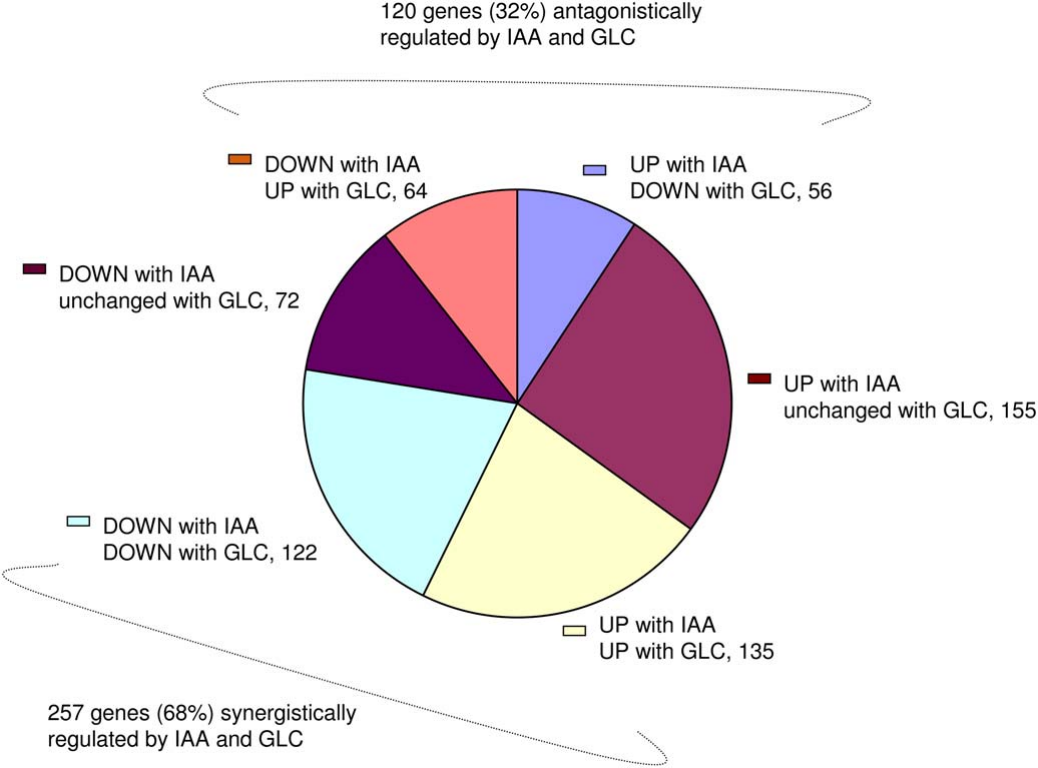
IAA up or down-regulated genes also affected 2 fold or more up or down by glucose treatment alone. Effect of glucose on IAA induced or repressed genes. IAA can altogether up or down-regulate 604 genes in glucose free medium (cut-off 2 fold) of which glucose alone can independently affect 377 (62%) genes (cut-off 2 fold).

In our observation, there were total 604 genes affected 2 fold up or down by IAA. Altogether, glucose could affect (2 fold or more) 377 (62%) out of total 604 genes significantly affected by auxin alone. Almost 257 (68%) genes, out of IAA affected 376 genes were agonistically regulated by glucose and rest 120 (32%) genes were antagonistically regulated by glucose (Figure 3; Figure S2). This is a huge number of genes simultaneously affected by sugar and auxin and may account for a number of common responses shared by sugar and auxin.

Out of total 346 genes up-regulated by IAA, 191 (55%) were also up or down-regulated by glucose alone. Out of 191 genes, 135 (71%) genes up-regulated by auxin are also up-regulated by glucose and rest 56 (29%) genes are antagonistically regulated i.e. down-regulated by glucose. Out of 258 down-regulated genes by IAA, 186 (72%) genes were also affected by glucose alone suggesting glucose alone can more extensively regulate IAA down-regulated genes (72%) as compared to IAA up-regulated genes (55%). Out of 186 IAA down-regulated genes, glucose could down-regulate 122 (65%) genes and 64 (34%) genes were antagonistically regulated by glucose (Figure 3; Figure S3).

**Figure 4:**
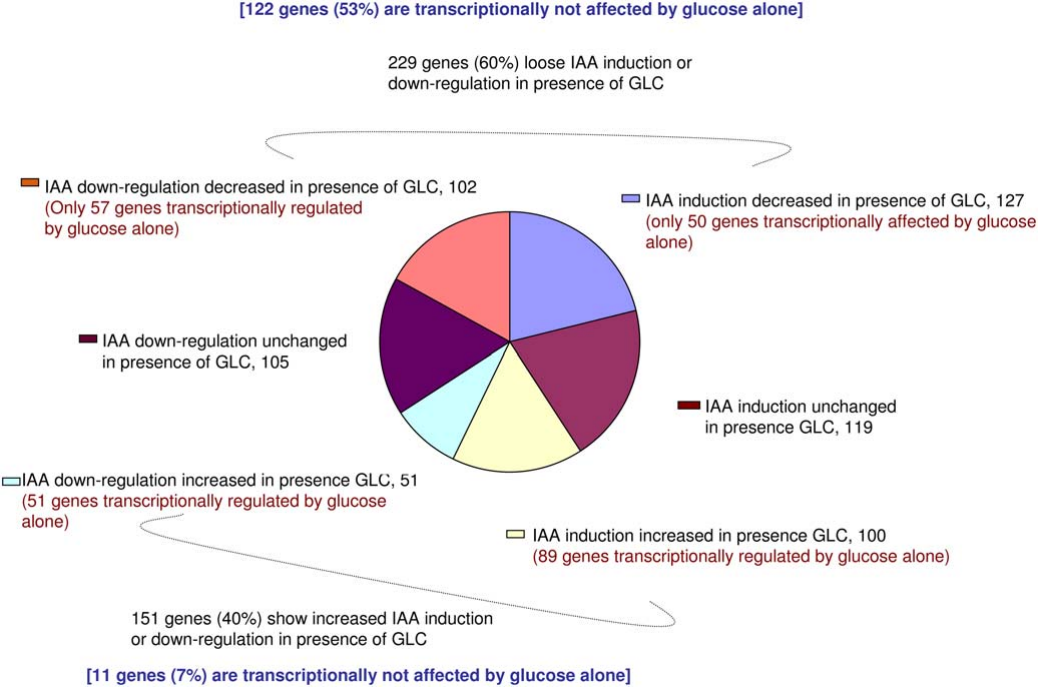
IAA up or down-regulated genes whose IAA-regulation was either lost or modulated up or down 2 fold or more on simultaneous glucose treatment. Effect of presence of glucose on the extent of IAA up-regulation or down-regulation of IAA affected genes in glucose free medium. Presence of glucose can change the extent of IAA induction or repression more then 2 folds for almost 63% IAA affected genes. Glucose can also affect IAA regulation of those genes which are themselves not regulated transcriptionally by glucose alone.

The other aspect we looked for was to find out how is the IAA induction or suppression of IAA related genes affected in presence of glucose in the medium. For that, the expression of all the genes in which either IAA mediated up-regulation or down-regulation of genes was affected 2 fold (up or down) or more in the presence of glucose in the medium was observed. Based on this criterion, 229 (60%) genes loose IAA mediated induction or down-regulation in presence of glucose, while 151 (40%) genes increase IAA mediated induction or down-regulation in the presence of glucose. Further, the induction of 227 (66%) of the 346 IAA up-regulated genes was either up-regulated or down-regulated more in the presence of glucose. Out of these 227 genes, IAA induction was decreased for 127 (56%) genes and increased for 100 (44%) genes in the presence of glucose. Although 89 (89%) genes in which IAA induction was increased in presence of glucose were also affected by glucose alone, only 50 (39%) genes in which IAA induction was down-regulated in presence of glucose were also affected by glucose alone transcriptionally (Figure 4; Figure S4). Noticeably, this category of genes mainly involves members of auxin related gene family, members of LOB domain containing protein family and a number of expressed proteins with unknown function.

Repression of 153 (59%) of 258 IAA down-regulated genes was either increased or decreased on glucose treatment. Out of the IAA down-regulated 153 genes, 102 (67%) genes had decreased IAA mediated down-regulation, while 51 (33%) genes had increased down-regulation in presence of glucose. 51 (100%) genes out of 51 were themselves down-regulated by glucose, while only 57 (56%) genes out of 102 genes were significantly up-regulated by glucose alone. Altogether, only 7% genes in which presence of glucose increased IAA mediated induction or down-regulation were transcriptionally not regulated by glucose alone, while 53% genes in which glucose antagonize IAA mediated induction or down-regulation are transcriptionally not regulated by glucose alone. Altogether, almost 61% IAA up-regulated genes in which up-regulation is lost in presence of glucose and 44% IAA down-regulated genes in which down-regulation is lost in presence of glucose (Figure 4; Figure S5) are themselves transcriptionally not significantly affected by glucose alone. This is an interesting finding and suggests that antagonistic effect of glucose on IAA-regulated gene expression requires some auxin regulated factor.

**Figure 5:**
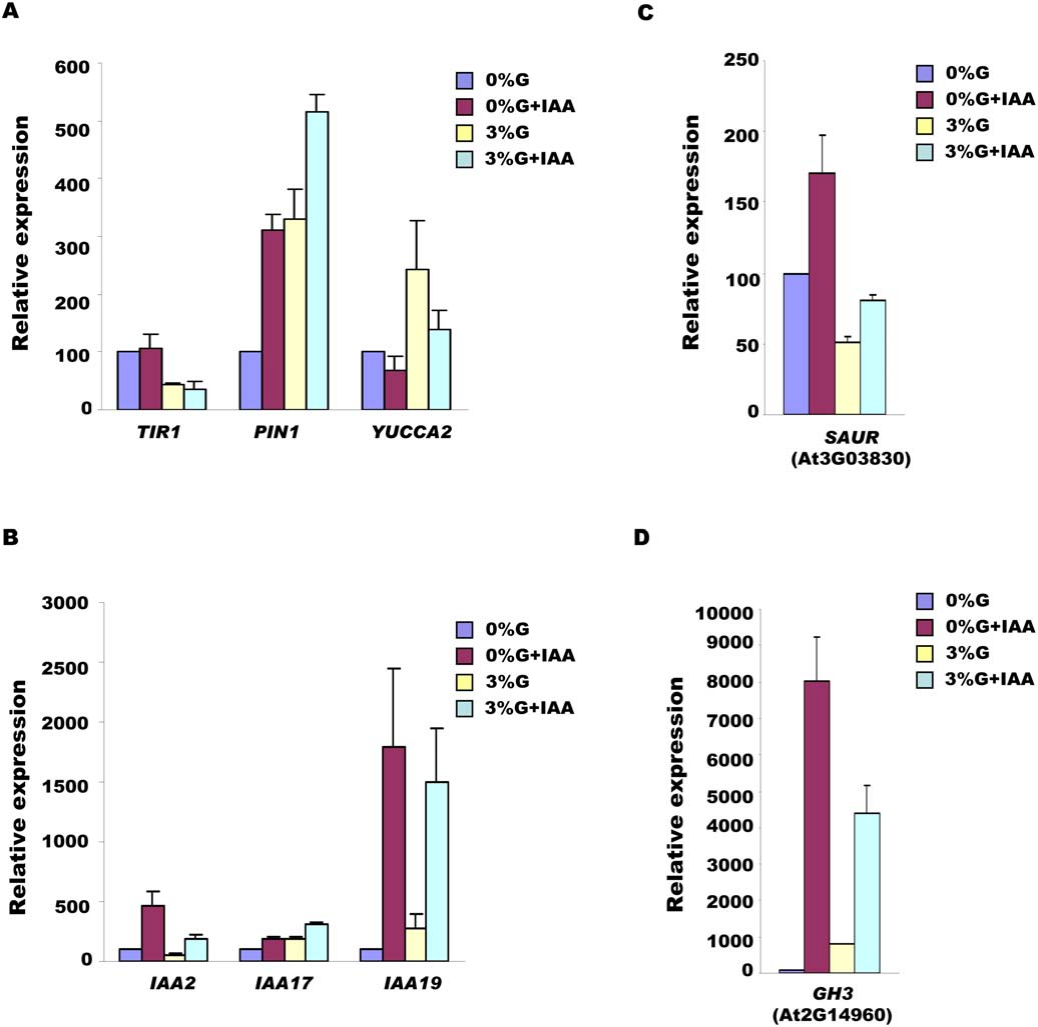
Quantitative Real –Time PCR validation of microarray results. The relative expression of a few representative genes from microarray data as revealed by Real time gene-expression analysis. 5 d old Col light-grown seedlings grown in 1/2MS medium containing 1% sucrose and 0.8% agar were depleted of sugars by placing them in sugar free 1/2MS liquid medium in dark for 24 h. The seedlings were then treated in dark with 1/2MS liquid medium containing 0%G, 0%G+1 µM IAA, 3%G and 3% G+1 µM IAA together for 3 h.

Briefly, glucose was found to affect almost all the important genes involved in auxin biosynthesis, perception, signaling and transport. *YUCCA2* involved in auxin biosynthesis was found to be up-regulated by glucose. At least two proposed auxin efflux gene family members including *PIN1* were up-regulated by glucose as two *ARF*s (Auxin Response Factors). Auxin receptor *TIR1* was found to be down-regulated by increasing concentrations of glucose while another proposed auxin binding protein and a receptor *ABP1* was found to be up-regulated by glucose. A number of genes involved in auxin signaling were either up-regulated or down-regulated by glucose. Altogether, 65% of glucose affected genes of SAUR, AUX-IAA, GH3 family genes were down-regulated by glucose (Figure S6). Relaxing the significance to 1.5 fold, 68 auxin related genes were found to be affected by glucose including auxin polar transporter *PIN2* and a number of AUX/IAA, SAUR and GH3 family members (Figure S7). The expression of auxin related genes *YUCCA2*, *TIR1*, *PIN1*, *IAA2*, *IAA17*, *IAA19*, *GH3* (AT2G14960) and *SAUR* (AT3G03830) was checked by doing real time PCR (Figure 5) confirming microarray results.

**Figure 6:**
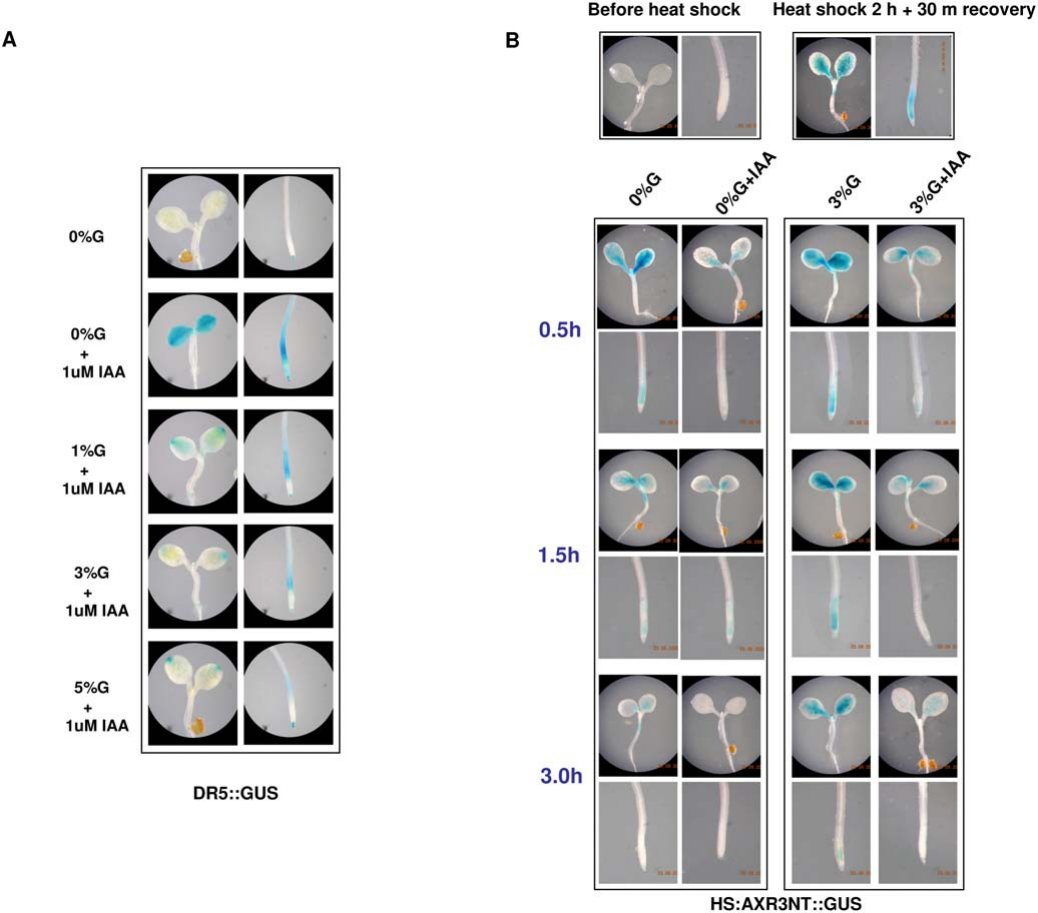
GUS expression analysis of DR5::GUS and HS:AXR3NT::GUS seedlings. (A) GUS expression in 5 d old light-grown DR5::GUS seedlings hypocotyls, cotyledons and roots treated for 3 h with different concentrations of glucose and IAA containing liquid 1/2MS medium. (B) Increasing glucose concentration increases the accumulation of (HS:AXR3NT::GUS) in 7 d old light-grown seedlings transferred to different concentrations of glucose containing liquid 1/2MS liquid medium for 0.5 to 3 h after 2 h heat shock followed by 30 m recovery. AXR3 is a repressor protein involved in down-regulating auxin signaling and itself gets degraded by auxin mediated proteasome pathway.

Auxin inducible promoter fused with GUS, DR5::GUS line was treated with different concentration of glucose and auxin. Seedlings treated with 0% glucose with 1 µM auxin showed an increase in GUS induction. Increasing the glucose concentration to 3% or 5% brought about a decrease in the auxin induced GUS expression both in the shoot and as well as in the roots (Figure 6A). To find out if glucose could modulate auxin signaling via affecting protein degradation, 7 d old HS:AXR3-NT::GUS [9] seedlings grown in regular MS medium were heat shocked for 2 h, left for recovery for 30 m and then transferred to different concentrations of glucose and auxin (IAA) containing medium for 3 h. The GUS expression as well as flurometry analysis suggested that the AXR3 protein is degraded more in 0%G containing medium while the presence of 3%G in the medium leads to less degradation of AXR3 protein in the seedlings at all the time points checked. IAA treatment led to degradation of AXR3 proteins both in 0%G and 3%G containing medium although some accumulation of AXR3 could still be noticed even after 3 h of degradation in 3%G containing medium (Figure 6B; Figure S8).

**Figure 7:**
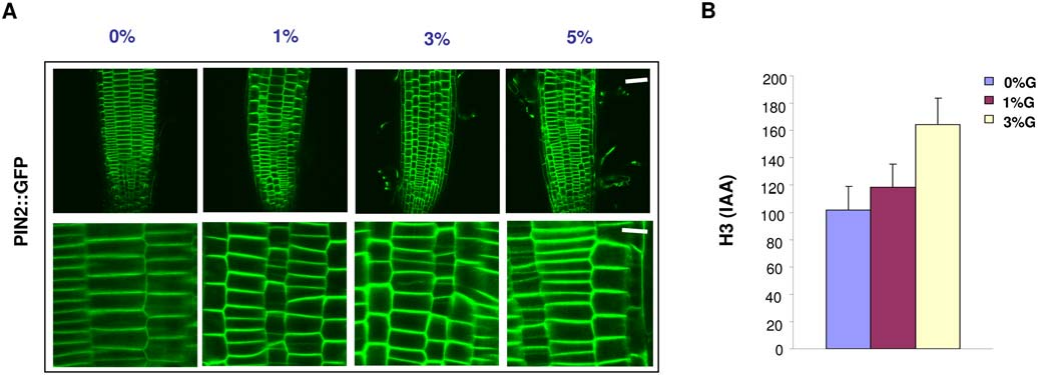
Accumulation and spatial expression of auxin transporter PIN2 as analyzed by PIN2::GFP expression in root tip by confocal microscope and root basipetal transport as measured using radio-labeled IAA. (A) PIN2::GFP expression in 5 d old light-grown seedlings *Arabidopsis* root tip treated for 4–5 h with different concentrations of glucose containing liquid MS medium. Increasing concentrations of glucose promotes more PIN2::GFP accumulation in the plasma membrane. Scale bar 50 µm for upper panel and 10 µm for lower panel. (B) Basipetal auxin transport increases on increasing glucose concentrations in 5 d old light-grown *Arabidopsis* seedling root tip as measured by H3(IAA) accumulation.

Since PIN proteins show a definite spatial-temporal expression and there partitioning in the cell is very important for determining the flow of auxin transport and thus the gravitropism, we also checked the expression of PIN2::GFP [10] proteins. On increasing concentration of glucose, the level of PIN2::GFP was also found to increase on both short term as well as long term treatment. In accordance, basipetal auxin transport was found to be more in seedlings treated with glucose suggesting that glucose signaling in fact does increase the auxin polar transport (Figure 7). A number of cells did show more accumulation of PIN2::GFP on the lateral walls on higher concentrations of glucose as compared to seedlings shifted to 0% glucose containing media.

**Figure 8:**
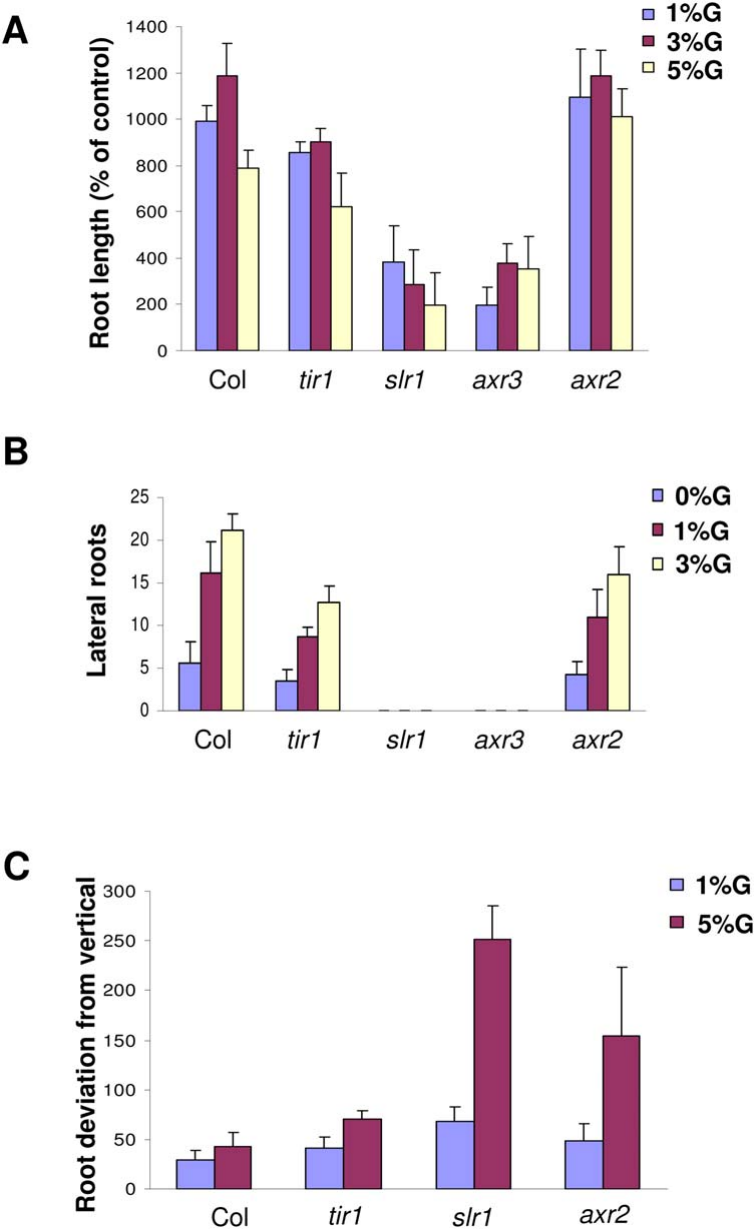
Comparison of root growth and development of auxin related mutants grown in different concentration of glucose containing medium. Effect of glucose on root length (A) lateral root induction (B) and root angle deviation from vertical (C) as demonstrated comparing 5 d old light-grown Col seedlings with different auxin receptor and signaling mutants. The Col and mutant seedlings were grown in 1/2MS medium containing 0.8% agar and 1% sucrose for 5 d to promote homogenous growth and then transferred to 1/2MS medium containing 0.8% agar with different concentrations of glucose for 3 d for root growth and root angle measurements and for 5 days for lateral root measurements.

**Figure 9:**
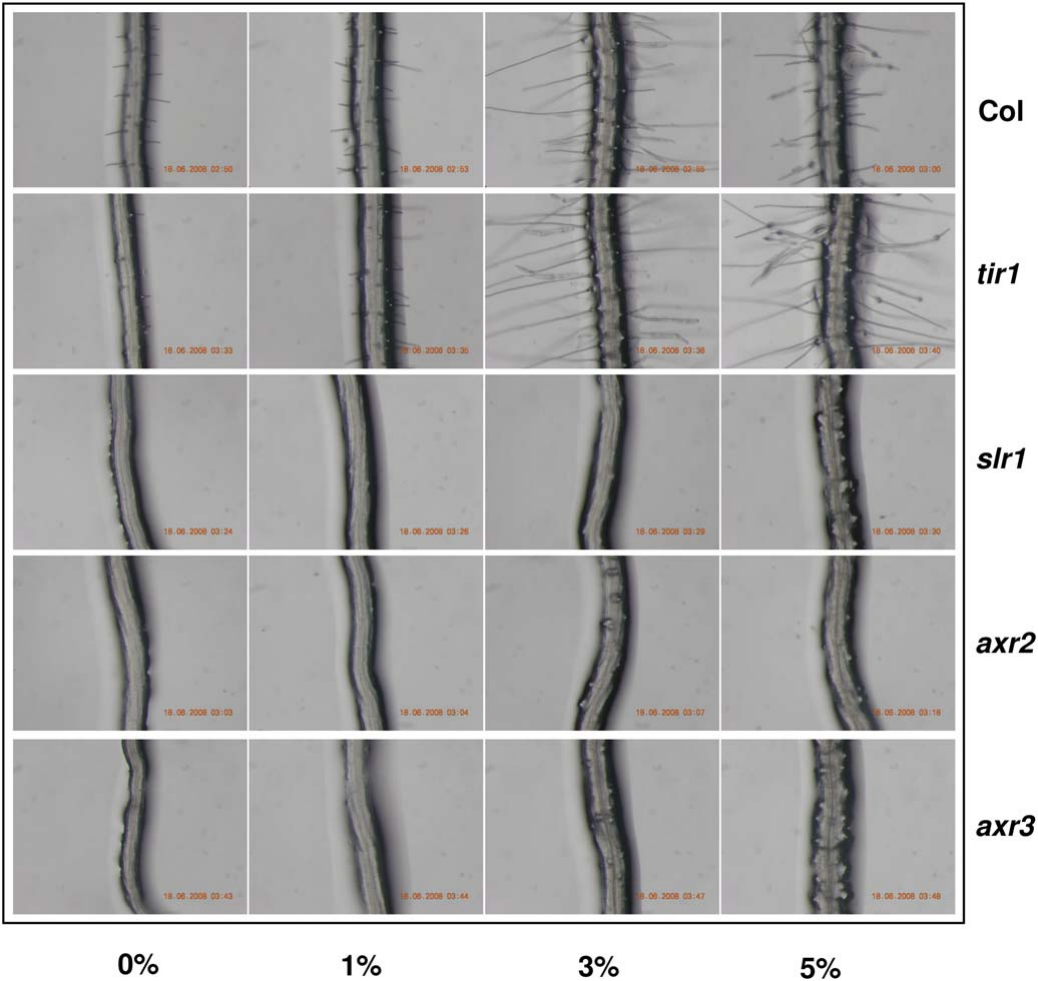
Comparison of root hair elongation of auxin response mutants grown in different concentration of glucose containing medium. Effect of glucose on root hair induction of 5 d old light-grown Col and different auxin receptor and signaling mutant seedlings. The Col and mutant seedlings were grown in 1/2MS medium containing 0.8% agar and 1% sucrose for 5 d to promote homogenous growth and then transferred to 1/2MS medium containing 0.8% agar with different concentrations of glucose for 3 d and photographs taken using a Nikon Coolpix camera attached to Nikon Stereo Zoom microscope.

To find out if glucose signaling is mediated by auxin response in controlling different root growth parameters and responses, we checked the effect of increasing concentrations of glucose on different auxin perception *tir1* and signaling *slr1/iaa14*, *axr3/iaa17* and *axr2/iaa7* mutants. Both root length change as well as lateral roots induction was significantly compromised in these mutants on increasing concentrations of glucose. The roots of auxin response mutant *tir1*, *axr2* as well as *slr1* are agravitropic in nature when grown in 1% glucose containing medium suggesting a role of proper auxin signaling required for optimal gravitropic response. On increasing concentration of glucose up to 5%, the roots growth of auxin signaling mutants becomes completely randomized with roots starting to go against gravity vector in *slr1/iaa14* and *axr2/iaa7* mutants (Figure 8; Figure S9) suggesting increasing concentration of glucose can accentuate the growth defect caused by perturbed auxin response/transport. These results suggest that glucose affects different root growth parameters via affecting auxin signaling/transport components. Glucose although could cause root hair initiation in auxin signaling mutant at high concentrations but root hair elongation was severely compromised (Figure 9) suggesting the requirement of normal auxin physiology a prerequisite even for this glucose induced parameter of root growth and development.

## Discussion

The nutrient status of the plant is very important as it has a profound effect on plant growth and development. Nutrients have been shown to work with hormones to modulate plant growth and development. In literature, there are several reports of interaction of sugar-response pathways with many other plant response pathways like nutrients, such as nitrogen, environmental-response pathways, such as those involved in light-response and hormone response. Although, there are several reports existing about sugar and phytohormone interaction [5], [11]–[14] but there are only very few reports on the nature of interaction between sugar and auxins. Auxin is one of the most important hormones regulating almost every aspect of plant growth and development from root development to control phyllotaxy and origin of a new branch. It also plays a very important role in response to changing environment such as light, gravity, and bacterial infection. Since a number of common responses are regulated by sugar and auxin, the obvious question arising is whether sugar and auxin act independently or interdependently to bring about changes in plant development and morphology/architecture. The possible link of sugar with auxin was revealed by the study of Hexokinase, *gin2* mutant. While *gin2* mutant hypocotyl explants are relatively insensitive to auxin-induction of cell proliferation and root formation, they were hypersensitive to shoot induction by cytokinin. Consistent with this observation, seedling development of the auxin-resistant mutants *auxin resistant1* (*axr1*), *axr2*, and *transport inhibitor response1* (*tir1*), and plants with a constitutive cytokinin response or supplemented with exogenous cytokinin is insensitive to high glucose levels [6]. Other reports of sugar auxin interaction comes from the study of turanose-insensitive mutant *tin* which was found to be encoding for WOX5 gene which is both turanose and auxin inducible. Moreover, *tin* mutant shows constitutive activation of indole-acetic-acid conjugation and *SUR2* expression. The role of WOX has been assigned in root apical meristem as a negative trigger of IAA homeostatic mechanism helping correct auxin maxima and root formation pattern [7]. The recent report in this field is the finding of a new allele of *hls1* (*h*ook*l*es*s*) mutant earlier isolated as an integrator of light, ethylene, and auxin signal transduction. This mutant has now been shown to be resistant to sugar and auxin responses, simultaneously, confirming the existence of a strong correlation between sugar and auxin signaling [8].

In the above mentioned literature, there are no systematic studies have been done which explore the global effect of glucose on auxin signaling and in turn plant growth and development. Further, there are no previous reports which suggest the possible effect of glucose on plant root gravitropism. In our study, we have found that increasing concentrations of glucose not only cause an increase in the root length, lateral roots and root hair production but also bring about more randomization in the direction of root growth.

The study of *gin2* mutant further suggested these root growth parameters and responses to be dependent on hexokinase mediated signaling to different extents. The *gin2* mutant also showed a constitutive high deviation from vertical in terms of root direction, suggesting normal product of *GIN2* is absolutely required for maintaining proper orientation of roots or buffers agravitropism. Glucose insensitive *gin2* mutant (defective in glucose signaling) as well as exogenous glucose application led to increased deviation from vertical suggesting that an optimal glucose signaling is absolutely required for proper gravitropism. A sub- or supra-optimal glucose signaling may perturb the normal gravitropic response exhibited by the wild-type seedlings. This may be due to a biphasic response of wild-type seedlings to both low and high concentration of glucose. It is worth mentioning here that a number of auxin-mediated responses have previously been documented to be biphasic [15], [16].

Microarray data analysis suggested that glucose can extensively affect auxin regulated gene expression. Almost 62% of the genes either up or down-regulated by IAA were also affected by glucose which includes 72% IAA down regulated genes and 55% IAA induced genes suggesting glucose can more extensively regulate IAA suppressed genes then induced genes. Almost 68% genes out of IAA affected 377 genes were agonistically regulated by glucose and 32% genes were antagonistically regulated by glucose suggesting a major crosstalk between the two signals. This is a huge number of genes simultaneously affected by sugar and auxin and may account for a number of common responses shared by sugar and auxin.

The other interesting observation emerging out of this study is that presence of glucose can affect the IAA up or down-regulation of almost 63% genes. The interesting observation is that glucose can even affect the induction or suppression of those IAA regulated genes which are themselves significantly not affected by glucose alone. Altogether, only 7% genes in which presence of glucose increased IAA mediated induction or down-regulation were transcriptionally not regulated by glucose alone, while 53% genes in which glucose antagonize IAA mediated induction or down-regulation are transcriptionally not regulated by glucose alone. Induction of almost 61% of IAA up-regulated genes and suppression of 44% IAA down-regulated genes is lost in the presence of glucose even though glucose alone could not affect these genes when present alone in the medium in absence of auxin. Noticeably, this category of genes mainly involves members of auxin related gene family including a number of AUX/IAA genes, members of LOB domain containing protein family and a number of expressed proteins with unknown function. Auxin related gene family has earlier been implicated in mediating auxin signaling extensively and controlling almost all the processes from embryogenesis to flowering [17]. LOB encodes a novel, plant-specific protein that is not similar to any proteins of known function. The LOB protein contains a conserved LOB domain which is also found in 42 other *Arabidopsis* proteins. LOB gene is expressed at the leaf base in a domain that defines a boundary between the meristem and the leaf. This expression pattern suggests a role in boundary establishment and organ separation, events that are critical for proper leaf development. Although, mutations in LOB genes do not cause conspicuous morphological changes, expression of LOB outside of its normal domain has several morphological effects [18]. This suggests that glucose may extensively modulate the plant growth and development by modulating response of auxin responsive genes to auxin or without directly affecting them at transcriptional level. This is an important observation and indicates that either an auxin dependent factor is required for sensitivity of these genes to glucose or in presence of auxin in the medium, glucose may be modulating some factor involved in auxin–regulated gene expression by some pathway not involving direct transcriptional changes of the affected genes. Here, it is important to mention that auxin signaling involves a number of auxin-regulated genes AUX/IAA encoding for proteins which negatively feedback their own regulation. These repressor proteins are degraded in presence of auxin by proteasome mediated pathway thus releasing their repressive effect. Glucose may modulate the stability of those proteins and thus can indirectly affect auxin regulated gene expression indirectly via non transcriptional pathways. In fact, glucose was found to affect degradation of auxin repressor protein AXR3 in HS:AXR3NT::GUS transgenic line. AXR3 gene encodes for one of the repressors in auxin signal transduction pathway whose degradation by 26S proteasome is increased on auxin application. In our study, this protein was found to be more stable in the presence of increasing concentrations of glucose suggesting glucose can stabilize at least some of the repressors of auxin signal transduction pathway. Since AXR3, a member of AUX/IAA gene family act as a repressor of auxin signaling; accumulation of it in the glucose treated seedling may possibly be one of the factors responsible for less induction of auxin induced gene on simultaneous glucose treatment. Altogether, these results suggest that glucose treatment may inhibit the activity of either proteasome complex itself or modulate the affinity of AUX/IAA proteins towards proteasome complex or its intermediate component, bringing about its less degradation. This a subject to further investigation though to find out how does glucose help AUX/IAA protein stabilization by protecting them from proteasome mediated degradation. An earlier report by Jen Sheen's group suggests negative role of sugar in controlling ethylene inducible transcription factor EIN3 by proteasomal degradation [19]. This observation thus carries a lot of significance and implies that sugar may modulate proteasome mediated degradation differentially in response to different stimuli.

Microarray studies as well as real time gene expression analysis suggests that increasing concentrations of glucose leads to an increase in the expression of auxin biosynthetic YUCCA gene family members. This increase is auxin biosynthesis though is not reflected at auxin induced gene expression level since glucose at the same time brings down the expression of auxin receptor TIR1. In fact, not only the expression of auxin receptor TIR1 was reduced, the auxin induction of a number of auxin inducible genes was also found to be reduced on exogenous glucose application. It is interesting to see the up-regulation of another proposed auxin receptor ABP1 at the same time which is mainly responsible for controlling cell expansion and elongation via controlling membrane transport processes in responses to auxin. Thus glucose may differentially modulate different receptors involved in auxin signaling controlling different physiological responses via different mechanisms of action.

The transcription of auxin transporter genes as well as auxin transport protein levels and rate of auxin transport was found to be more in glucose treated seedlings suggesting glucose may modulate auxin transport by increasing the accumulation of auxin transporter proteins on the plasma membrane. Glucose could also alter the spatial expression of PIN2::GFP in terms of its more accumulation in the lateral walls. This may lead to more lateral auxin flux and would correlate with the more lateral root production on high concentration of glucose. Earlier, different light conditions as well as dark have also been reported to affect PIN2::GFP partitioning between plasma membrane and vacuoles affecting root growth and development [20].

Increasing concentrations of glucose on different auxin perception *tir1* and signaling *slr1/iaa14*, *axr3/iaa17* and *axr2/iaa7* mutants induce a differential response in terms of root growth and lateral roots induction. Since auxin signaling and different polarities of auxin transport prominently regulate lateral root formation in *Arabidopsis* seedlings, perturbed auxin homeostasis in auxin mutant may account for resistance to glucose treatment. On increasing concentrations of glucose, the roots growth of auxin signaling mutants becomes completely randomized with roots starting to go against gravity vector in *slr1/iaa14* and *axr2/iaa7* mutants suggesting increasing concentration of glucose can accentuate the growth defect caused by perturbed auxin signaling as present in *slr1* and *axr2* mutant. Glucose may either accentuate the growth defects of auxin signaling mutants by further disrupting auxin signaling or via modulating auxin transport. Gravitropism is perceived by the amyloplasts in the columella cells from where the signal is transmitted to the zone of cell elongation where asymmetric accumulation of auxin takes place. Asymmetric auxin distribution in turn leads to differential growth and gravitropic response. There are other reports wherein different stimuli such as salt, hydrotropism and light may affect gravitropic responses by modulating auxin signaling/transport machinery. Salt has been shown to disrupt not only amyloplasts accumulation but also PIN2 accumulation and orientation [21]. In our observation the higher concentration of glucose does not cause disruption in amyloplasts formation (data not shown) but it causes perturbed auxin signaling and an increased accumulation of PIN2::GFP on the membrane translating to increased auxin transport. This suggests that both glucose and salt may be employing different mechanisms to affect plant root gravitropism and glucose primarily depending on auxin signaling/transport in controlling root gravitropic response.

Increasing concentrations of glucose could although promote initiation of root hair formation but the root hair could not elongate normally in the auxin related mutants suggesting a major role of auxin signaling in mediating glucose induced root hair elongation. High concentration of glucose could rescue root hair initiation in root hairless mutants like *slr1/iaa14*, *axr2/iaa7* and *axr3/iaa17* suggesting that high glucose concentration is able to overcome the repression of root hair initiation maintained by *iaa* gain-of-function mutation where mutated IAAs have an increased stability and can not be turned over by proteasome pathway. This may mean that high glucose levels destabilize mutated AUX/IAA protein in a way that differs from IAA mediated Aux/IAA destruction or that high glucose induces the accumulation of a transcription factor which can shift Aux/IAAs and make ARF free to induce transcription of auxin regulated genes. High glucose concentration might also activate stress related pathway antagonizing the affect of AUX/IAA mutated proteins.

Our studies suggest that glucose can affect almost all aspects of auxin metabolism from auxin biosynthesis to transport, perception and signaling leading to altered plant growth and development. Glucose not only affects auxin-regulated gene expression but may also control some non transcriptional processes such as protein stability/degradation to ultimately affect auxin mediated signaling and responses. Further dissecting how precisely the antagonistic and agonistic interaction between glucose and auxin signaling are controlled is a subject to further study. Moreover, finding out the precise molecular mechanism of specificity involved in glucose control of protein degradation also remains a major challenge. A detailed analysis of developmental, tissue specific and temporal regulation of glucose-auxin interaction would shed further light on how these two very important signals integrate to control plant growth and development in broader context.

## Methods

### Materials and Growth Conditions

All seed stocks were obtained from the Arabidopsis Biological Resource Center at Ohio State University except that PIN2::PIN2-eGFP, DR5::GUS and HS::AXR3NT-GUS lines were obtained from NASC stock centre.

Seeds were surface sterilized and imbibed at 4°C for 48 h. The imbibed seeds were germinated and grown vertically on Petri dishes containing 0.5× Murashige and Skoog (Sigma) supplemented with 1% sucrose and solidified with 0.8% Agar (Hi Media). Seed germination was carried out in climate-controlled growth rooms in a long day condition (16 hr light and 8 hr darkness), except stated otherwise, with 22°C±2°C temperature and 80 µmol/sec/m^2^ light intensity. All chemicals were from Sigma except specified otherwise, and prepared as DMSO stock solutions.

### Microarray analysis

*Arabidopsis thaliana* seeds (ecotype Columbia-0) were surface sterilized and water imbibed in the dark for 3 d at 4°C. The seeds were then inoculated in 1/2MS medium supplemented with 0.8% agar and 1% sucrose. Once the plant material was uniformly germinated, the experimental conditions were applied. 5 d old light-grown uniformly germinated seedlings were washed seven times with sterile water with last wash given by 1/2MS liquid medium to remove residual exogenous sugar and the plant material was kept in 1/2MS liquid without sucrose in the dark for all subsequent steps. Cultures were shaken at 140 rpm at 22°C for 24 h and then 3 h treatment was given with liquid 1/2MS without glucose and liquid 1/2MS supplemented with IAA (1 µM), glucose (3%), glucose (3%)+IAA (1 µM). Seedlings were harvested after 3 h and preceded for RNA isolation and Microarray analysis. RNA was prepared from frozen tissue using the RNeasy kit (Qiagen, Valencia, CA) following the manufacturer's protocol. The RNA was quantified and tested for quality before it was used for subsequent analyses. Three biological replicates were used for doing Microarray analysis. Labeling of RNA Probe and Hybridization to Arabidopsis Gene Chip Labeling and hybridization of RNA were conducted using standard Affymetrix protocols by the University of California, Irvine DNA MicroArray Facility. Briefly, ATH1 Arabidopsis GeneChips (Affymetrix, Santa Clara, CA) were used for measuring changes in gene expression levels. Total RNA was converted into cDNA, which was in turn used to synthesize biotinylated cRNA. The cRNA was fragmented into smaller pieces and then was hybridized to the Gene Chips. After hybridization, the chips were automatically washed and stained with streptavidin phycoerythrin using a fluidics station. The chips were scanned by the Gene Array scanner by measuring light emitted at 570 nm when excited with 488-nm wavelength light. Data from the Gene Chip experiments were analyzed using ArrayAssist (Stratagene). Briefly .CEL files of 3 biological replicates with correlation>0.95 were used for experimental grouping. Expression values were normalized using GC-RMA algorithm. The data was then Log transformed and replicates were averaged. Differential gene expression analysis was done for all the treatment *vs* control. A two fold cut-off at a Pval of 0.05 was employed to find out significant genes. Additional microarray data presentation and manipulation were assessed using Microsoft Excel.

### Measurement of root length, lateral root, root hair and gravitropic responses

Five day-old seedlings grown vertically on ½ MS 0.8% agar and 1% sucrose containing medium were transferred to ½ MS 0.8% agar containing medium with different concentrations of glucose and their root tips marked. Digital images of root tip were captured after 48 h. Changes in root tip curvature and root length were quantified using the Image J program from NIH. Lateral root were quantified by counting directly under Nikon StereoZoom microscope after 5 d of transfer. Root hair photographs were taken by Nikon Coolpix camera fitted with Nikon StereoZoom microscope. The data was the average of 10seedlings±standard deviation.

### Gene expression analysis -Real time PCR

Real time PCR reactions were carried out using the same RNA samples, which were used for microarrays as described earlier. In brief, primers were designed for all the genes preferentially from 3′ end of the gene using PRIMER EXPRESS version 2.0 (PE Applied Biosystems, USA) with default parameters. First strand cDNA was synthesized by reverse transcription using 4 µg of total RNA in 100 µl of reaction volume using high-capacity cDNA Archive kit (Applied Biosystems, USA). Diluted cDNA samples were used for Real time PCR analysis with 200 nM of each primer mixed with SYBR Green PCR master as per manufacturer's instructions. The reaction was carried out in 96-well optical reaction plates (Applied Biosystems, USA), using ABI Prism 7000 Sequence Detection System and software (PE Applied Biosystems, USA). To normalize the variance among samples, 18SrRNA was used as endogenous control. Relative expression values were calculated after normalizing against the maximum expression value. The values presented are the mean of the two biological replicates, each with three technical replicates. The error bars indicate standard deviation from the mean. The primer sequences for all the genes tested have been included in Figure S10.

### Laser Confocal Scanning Microscopy (LCSM)

GFP fluorescence was imaged under a Leica TCS SP2 AOBS Laser Confocal Scanning Microscope (Leica Microsystems, Exton, PA). For imaging GFP, the 488 nm line of the Argon laser was used for excitation and emission was detected at 520 nm. The laser, pinhole and gain settings of the confocal microscope were kept identical among different treatments. Images were assembled using Photoshop (Adobe Systems).

### GUS histochemical staining and flurometry

DR5::GUS and HS:AXR3NT::GUS reporter was determined using a standard GUS histochemical staining procedure. Briefly, 5-day-old DR5::GUS, seedlings grown in 1/2MS medium with 1% sucrose were transferred to different concentration of glucose and IAA containing medium for 3–4 h. GUS activity was then determined by incubating the seedlings at 37°C for 3–4 h. The 7 d old HS:AXR3NT::GUS seedlings grown in 1/2MS medium containing 1% sucrose were heat-shocked at 37°C for 2 hrs followed by recovery for 30 m to induce HS:AXR3NT::GUS reporter. Seedlings were subsequently incubated in room temperature in the growth medium supplemented with or without different concentrations of glucose for 1/2 h, 1.5 hr and 3 h. GUS activities were then determined by incubating the seedlings at 37°C in a GUS staining solution (Sodium phosphate buffer pH 7, 0.1 M, 0.5 mM K_3_Fe(CN)_6_, 0.5 mM K_4_Fe(CN)_6_, EDTA 50 mM, X-Gluc 1 mg/ml) for 10–12 hrs. The seedlings were then kept in 70% ethanol for the removal of chlorophyll. The seedlings were then observed under Stereo-Zoom microscope and photographs taken using Nikon Coolpix digital camera.

Flurometric assay was performed by homogenizing the samples in extraction buffer (50 mM NaPO_4_, pH 7.0; 10 mM β-mercaptoethanol; 1 mM Na_2_EDTA; 0.1% Sodium Lauryl Sarcosine; 0.1% Triton×100). Protein was quantified using Bradford Assay and data was normalized against total protein level. Samples were assayed using 1 mM MUG (4-methylumbelliferyl-β-D-glucoronide) in extraction buffer followed by incubation of 2–3 hrs. The reaction was stopped by adding 0.2 M Na_2_CO_3_ and the sample readings were taken with a Modulus Luminometer (Turner Biosystem).

### Auxin transport assay

Root basipetal auxin transport was measured essentially as previously described [22]. Briefly, agar blocks of 1 mm in diameter containing 7.7×10^−8^ M ^3^H-IAA (Amersham) was applied at root tip. After incubation for 1.5 hrs, a 0.5 mm section of the root tip close to the agar block was dissected and discarded. Two consecutive 2-mm segments above the incision line were then collected separately and pooled from 6 to 10 roots and placed into glass scintillation vials containing 5 mL scintillation fluid. Radio-activities in these two pools of root segments were measured using a Beckman Coulter LS6500 Scintillation counter (Fullerton, CA, USA). The amount of the radioactivity was the average of three separate experiments±standard deviation. Student's *t*-test with paired two-tailed distribution was used for statistical analysis.

## Supporting Information

Figure S1 Effect of non signaling glucose analog 3-OMG on 5 d light-grown Col seedlings transferred to different concentrations of glucose and 3-OMG containing MS medium for 3 d.
(1.53 MB TIF)

Figure S2 List of IAA up-regulated genes also affected 2 fold or more up or down by glucose treatment alone.
(0.09 MB XLS)

Figure S3 List of IAA down-regulated genes also affected 2 fold or more up or down by glucose treatment alone.
(0.12 MB XLS)

Figure S4 List of IAA up-regulated genes whose IAA-induction was either lost or modulated up or down 2 fold or more on simultaneous glucose treatment.
(0.33 MB XLS)

Figure S5 List of IAA down-regulated genes whose IAA-repression was either lost or modulated up or down 2 fold or more on simultaneous glucose treatment.
(0.12 MB XLS)

Figure S6 List of IAA-related genes involved in IAA biosynthesis, transport, perception and signaling affected 2 fold or more on glucose treatment.
(0.02 MB XLS)

Figure S7 List of IAA-related genes involved in IAA biosynthesis, transport, perception and signaling affected 1.5 fold or more on glucose treatment.
(0.03 MB XLS)

Figure S8 GUS flurometric analysis of HS:AXR3NT::GUS seedlings to show quantitatively more accumulation of AXR3 protein in 3% glucose containing MS medium.
(1.46 MB TIF)

Figure S9 Root phenotype and tropic response of auxin related mutants, tir1, axr2, axr3 and slr1 on 1% and 5% glucose containing medium.
(13.13 MB TIF)

Figure S10 List of primers used for validating microarray data doing real-time gene expression analysis.
(0.02 MB XLS)
