# Supplementary figures and images for: Glucose and Auxin Signaling Interaction in Controlling Arabidopsis thaliana Seedlings Root Growth and Development
Source: PLoS One. 2009 Feb 18;4(2):e4502. doi: 10.1371/journal.pone.0004502 (PMC2637607; doi:10.1371/journal.pone.0004502)

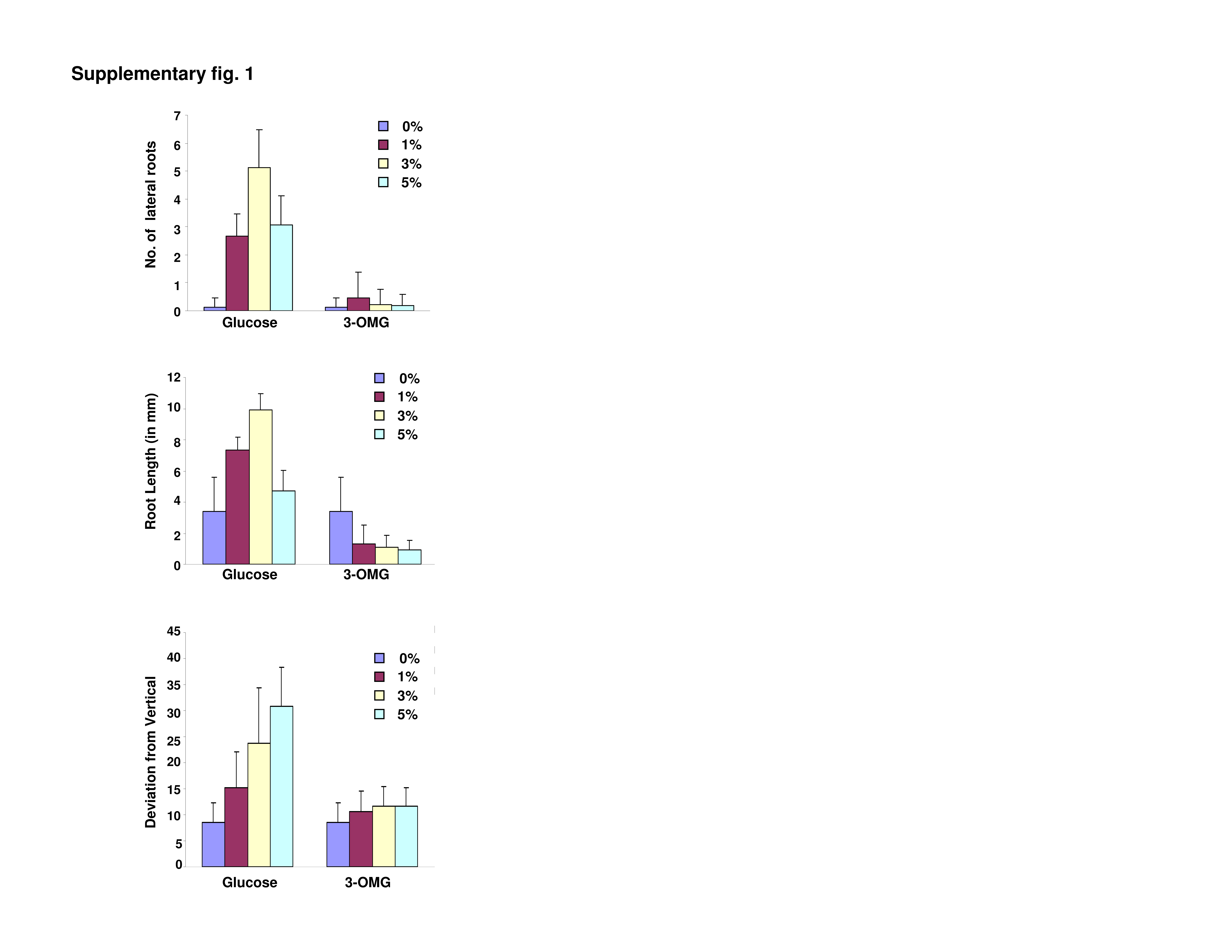

Supplement: Figure S1 — Effect of non signaling glucose analog 3-OMG on 5 d light-grown Col seedlings transferred to different concentrations of glucose and 3-OMG containing MS medium for 3 d. (1.53 MB TIF) [file pone.0004502.s001.tif]

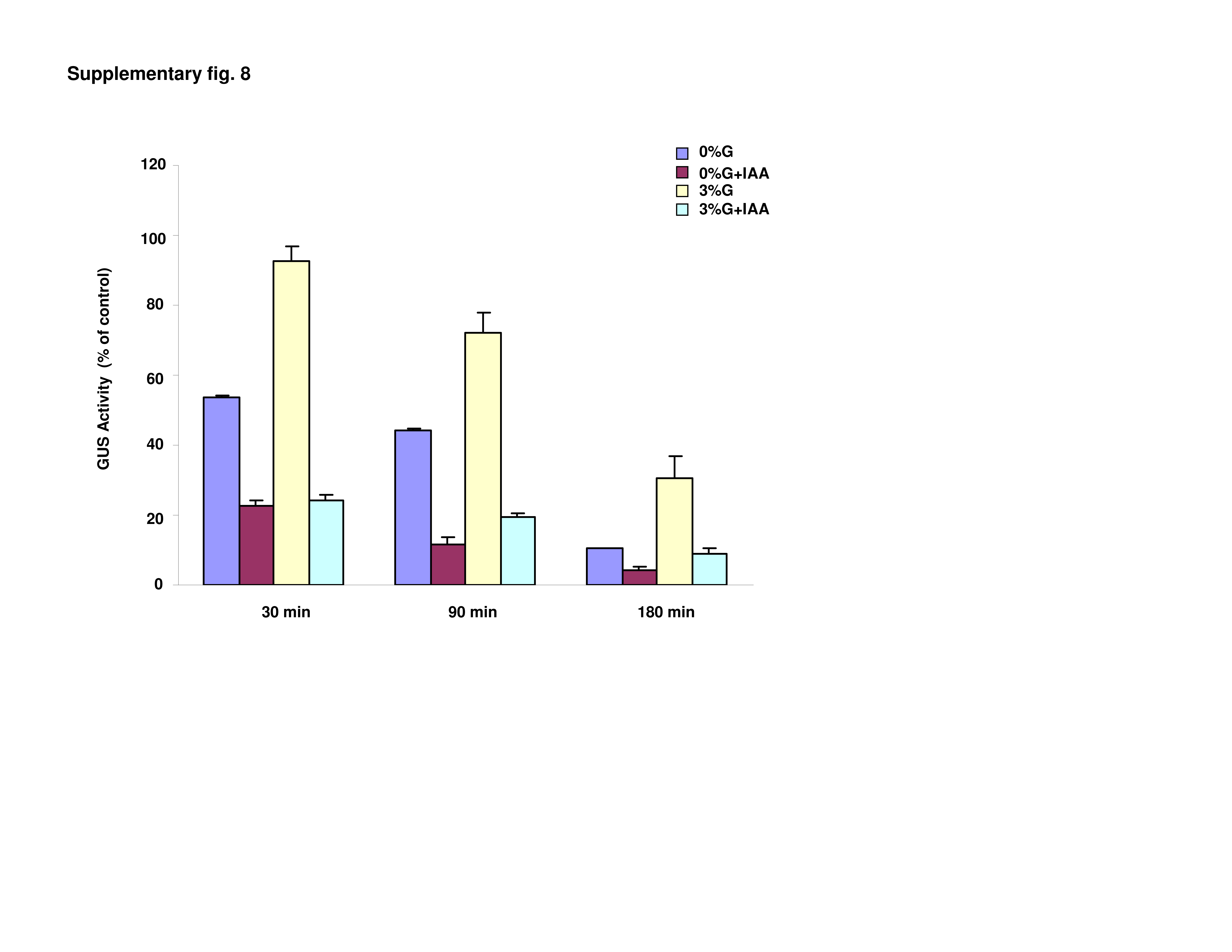

Supplement: Figure S8 — GUS flurometric analysis of HS:AXR3NT::GUS seedlings to show quantitatively more accumulation of AXR3 protein in 3% glucose containing MS medium. (1.46 MB TIF) [file pone.0004502.s008.tif]

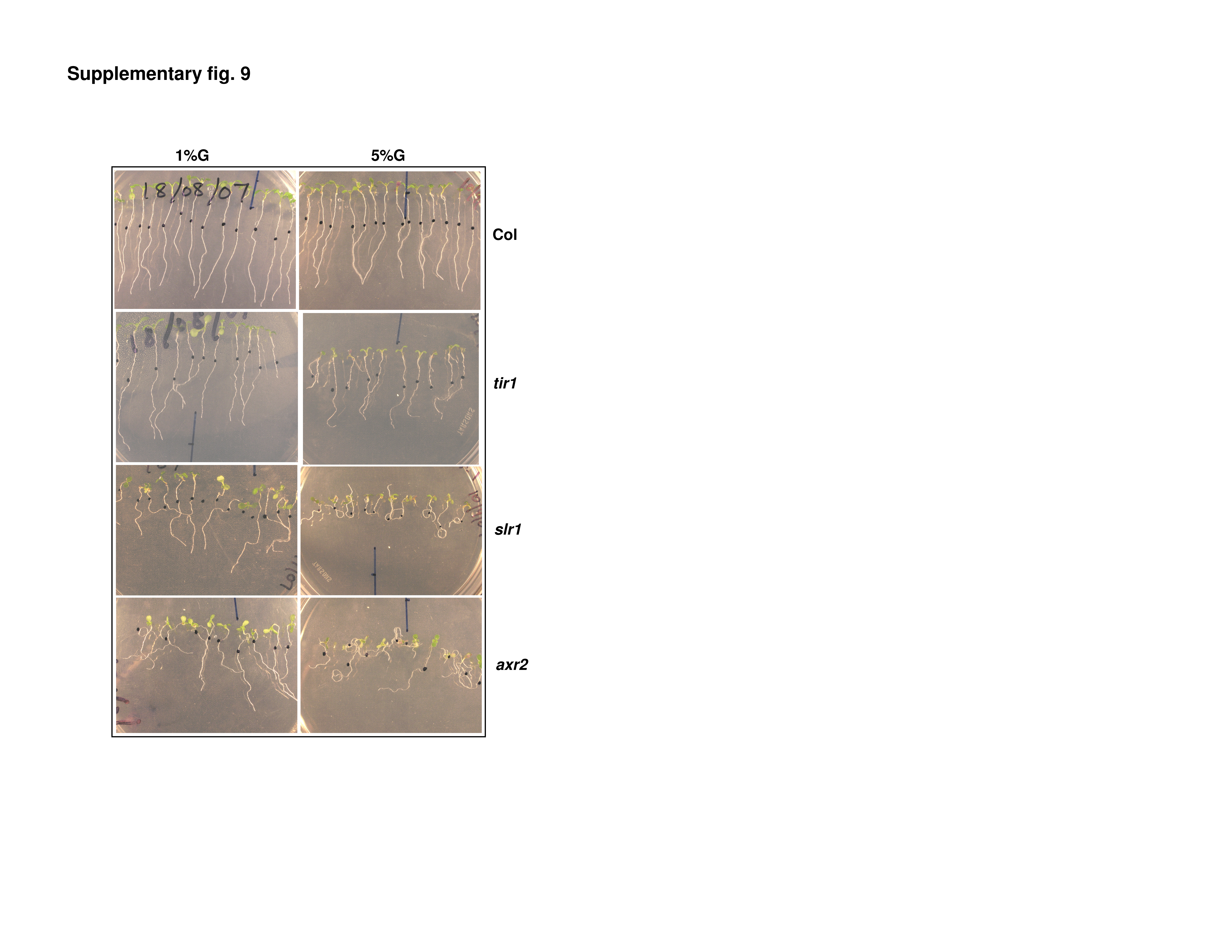

Supplement: Figure S9 — Root phenotype and tropic response of auxin related mutants, tir1, axr2, axr3 and slr1 on 1% and 5% glucose containing medium. (13.13 MB TIF) [file pone.0004502.s009.tif]
